# Microbial Contamination after Cardiac Surgery in a Hospital Cardiac Surgery Ward

**DOI:** 10.25122/jml-2019-0071

**Published:** 2020

**Authors:** Donya Shahedi Damavandi, Mina Javan, Hamidreza Moshashaei, Mojgan Forootan, Mohammad Darvishi

**Affiliations:** 1. Faculty of Medicine, Tehran Medical Sciences Branch, Islamic Azad University, Tehran, Iran; 2. Department of Gastroenterology, Gastrointestinal and Liver Diseases Research Center (RCGLD),Shahid Beheshti University of Medical Sciences, Tehran, Iran; 3. Infectious Diseases and Tropical Medicine Research Center (IDTMRC), Department of Aerospace and Subaquatic Medicine,AJA University of Medical Sciences, Tehran, Iran

**Keywords:** Cardiac surgery, microbial contamination, incidence, type of infection, pathogen

## Abstract

Surgery site infection is one of the most common postoperative complications which is associated with increased morbidity, mortality and admission costs. It is considered a priority to determine the level of nosocomial infection and its control in reflecting the quality of care. Therefore, this study aimed to evaluate the microbial contamination after cardiac surgery at a hospital cardiac surgery ward of Besat Hospital, Tehran.

In this cross-sectional descriptive-analytic study (2013-2017), 610 patients underwent surgery at the Department of Cardiac Surgery of Besat Hospital. All necessary information such as urine culture, surgical site, histopathologic examination for the diagnosis of microbial contamination and microorganisms were collected from the patient records and inserted in the questionnaire. The data were analyzed using SPSS (version 25).

The incidence of nosocomial infections following cardiac surgery reportedly ranged from 17% to 23%. Accordingly, pneumonia (51.2%) and local infections (22%) were the most common infections in the studied population. The mortality rate in our population was 11.4%. Moreover, 64.3% of the total mortality cases were reported in patients with sepsis. The mean age and duration of admission of patients with catheter infection were significantly higher than other subjects.

Given the relatively high prevalence of the infection and its importance, it is necessary to take more serious measures to prevent and control these infections.

## Introduction

Nosocomial infections affect hospitalized patients. The manifestations may emerge during or after the discharge of the patient [1]. Usually, infections that emerge after 48 to 72 hours are considered as nosocomial infections [2]. Nowadays, due to increased mortality rates, increased duration of hospitalization, increased costs of prolonged admission, diagnostic and therapeutic measures, nosocomial infections are one of the critical medical problems [3]. In the United States (US), for every 100 hospital admissions, 5-7 cases of the nosocomial infection are reported. Therefore, at least 2.1 million hospitalizations occur annually in the country. It seems that in all parts of the world, nosocomial infection cases are as important as in the US [4]. The rate of nosocomial infections in Iran ranges from 1.9% to 25% [5]. Among the various types of infections in the hospital, urinary tract infection, lower respiratory tract infections or pneumonia, surgical site infections, and digestive tract infections account for 42%, 15-20%, 24% and 5-10% of infections, respectively [6]. Urinary tract infections are the most common infections, while pneumonia is the deadliest infection in the hospital. However, in some centers, nosocomial infection of the circulatory system is the main cause of death, and surgical infection is the second most common cause of nosocomial infections in hospitalized patients. Moreover, surgical site infection accounts for 25% of the infections [7]. 

The most common complication of cardiac surgery is infection. Over 600,000 patients undergo cardiac surgery annually all over the world. Accordingly, 47% lose their lives because of infections [7]. Nosocomial infections are a significant cause of complications and mortality after cardiac surgeries [8]. These infections can be superficial and include subcutaneous tissues. They may be deep, involving the sternum or mediastinum [9]. On average, 2-20% of patients undergo surgical site infections after cardiac surgery [10]. Surgery site infections, especially sternal and mediastinal infections, significantly increase morbidity, mortality, and costs [11]. 

In general, it can be said that controlling nosocomial infections in different sections of the hospital is now a priority in the world. It is necessary to minimize infections, in addition to reducing mortality, reducing the duration of admissions, and reducing the cost of treatment. Therefore, considering the importance of timely diagnosis and treatment of infection, it is essential to investigate microbial infections after cardiac surgery in cardiac surgery wards, and that is the primary purpose of this study.

## Material and Methods

In this cross-sectional study (2013-2017), the research population included all patients admitted to the cardiac surgery department of Besat Hospital.

### Sample size

Given that the rate of nosocomial infection has been reported differently in previous studies, so assuming p equals to 0.5 (the most unfortunate case), and according to the type I error (α=0.05) and accuracy (d=0.1), the minimum sample size for this study was approximately 100 patients according to the formula given below. At the time of our study, 123 cases of infection were reported, indicating a sufficient sample size to determine relationships.

The inclusion criteria were the following: all patients undergoing surgery at the Department of Cardiac Surgery of Besat Hospital (2013-2017), patients hospitalized for at least 48 hours in the Cardiac Surgery Department, and the onset of symptoms of the nosocomial infection after 48 hours of admission.


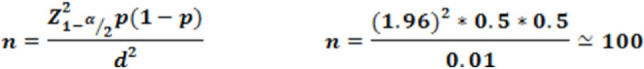


### Demographic data and medical records

Examination of urine culture, surgical infection site, phlegm or aspiration fluid through the chest wall, blood collection, evaluation of radiological reports based on evidence of abscess, infiltration, consolidation or pleural effusion, histopathologic examination for nosocomial infection detection and microorganisms were performed. Data regarding age, sex, body mass index (BMI), duration of admission and underlying disease, along with all ethical considerations, were collected and recorded in the questionnaires.

### Data analysis

The collected data were entered into the SPSS software (version 25) and analyzed by Proper statistical tests at a significance level of 0.05.

## Results

Of the total number of people with nosocomial infections, 80 were men (65%). The participants were aged between 38 and 85 years and the mean age was 62.11 ± 9.90 years. The majority of the participants were 51-70 years old (65.8%). Most of the participants were 51-60 years old (34.1%) and 61 to 70 years (31.7%).

The mean age of patients with catheter infection was significantly higher than the other subjects, with a mean age of 72.33 ± 11.03 years. Meanwhile, the mean age of the other groups was 9.59 ± 61.59 years (p-value = 0.009). However, there was no relationship between the other infections and the age of patients (Table 1).

**Table 1:** The relationship between patient age and nosocomial infections.

Group Statistics					
		N	Mean	Std. Deviation	p-value t-student
**SSI**	+	27	61.2222	10.89578	0.598
	-	96	62.3646	9.64869	
**Pneumonia**	+	63	63.7302	9.07532	0.64
	-	60	60.4167	10.50793	
**Sepsis**	+	18	61.1667	11.83340	0.66
	-	105	62.2762	9.58715	
**UTI**	+	22	62.5909	10.71563	0.804
	-	101	62.0099	9.76780	
**Catheter infection**	+	6	72.3333	11.03932	0.009
	-	117	61.5897	9.59989	
**SIRS**	+	6	64.0000	16.60120	0.634
	-	117	62.0171	9.54028	

Note: SSI - Surgical site infection, UTI - Urinary tract infections, SIRS - systemic inflammatory response syndrome.

According to the Chi-square test, there was no difference between the sex of the patients and postoperative nosocomial infection in the surgery department (p-value <0.05) (Table 2). Also, the distribution of subjects in terms of BMI showed that 42 (34.1%) had a normal BMI. Fifty-four patients (43.9%) were overweight, and 27 (22%) had obesity (Table 3).

**Table 2:** The relationship between the sex of the patients and the type of nosocomial infection.

**Crosstab**					
			**Sex**		**p-value chi-square**
			**Male**	**Female**	
**Sepsis**	+	Count	11	7	0.448
		%	13.8%	16.3%	
	-	Count	69	36	
		%	86.3%	83.7%	
**SSI**	+	Count	15	12	0.173
		%	18.8%	27.9%	
	-	Count	65	31	
		%	81.3%	72.1%	
**UTI**	+	Count	14	8	0.531
		%	17.5%	18.6%	
	-	Count	66	35	
		%	82.5%	81.4%	
**SIRS**	+	Count	5	1	0.314
		%	6.3%	2.3%	
	-	Count	75	42	
		%	93.8%	97.7%	
**Catheter infection**	+	Count	4	2	0.65
		%	5.0%	4.7%	
	-	Count	76	41	
		%	95.0%	95.3%	
**Pneumonia**	+	Count	42	21	0.421
		%	52.5%	48.8%	
	-	Count	38	22	
		%	47.5%	51.2%	

Note: SSI - Surgical site infection, UTI - Urinary tract infections, SIRS - systemic inflammatory response syndrome.

**Table 3:** Distribution of subjects by BMI.

**BMI**			
		**Frequency**	**Percent**
**BMI**	**Normal**	42	34.1
	**Overweight**	54	43.9
	**Obese**	27	22.0
	**Total**	123	100.0

**Figure 1:**
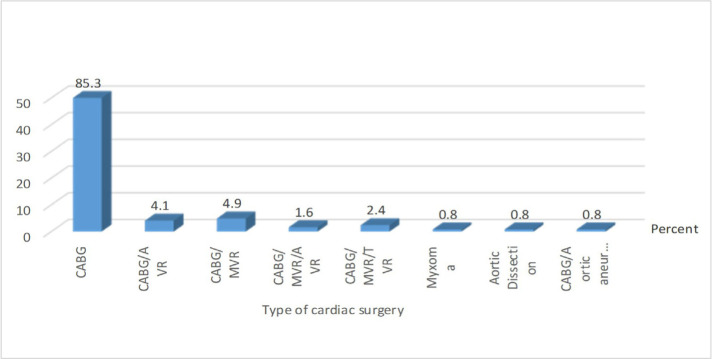
Frequency distribution of the subjects based on type of cardiac surgery.

The study of the distribution of the subjects according to the type of cardiac surgery showed that cardiopulmonary bypass surgery was the most common type of surgery among the subjects. Accordingly, other types of surgeries accounted for only 1.6% of surgeries (Figure 1).

The distribution of the subjects in terms of the surgery site (sternum) revealed that in 4 patients (3.3%), the postoperative surgery site was left open. According to the results, 120 (97.6%) patients had other diseases, of which hypertension (64.2%), coronary artery disease (61.8%), diabetes (43.9%) and hyperlipidemia (35.8%) were the most common (Table 4). The rates of leukocytosis, erythrocyte sedimentation rate (ESR), C-reactive protein (CRP) and serum proxlotonin levels were 87%, 75%, 85.5% and 38.2%, respectively.

**Table 4:** Distribution of subjects based on the type of the disease.

	Frequency	Percent
**CAD (Coronary artery disease)**	76	61.8
**DM (Diabetes mellitus)**	54	43.9
**HLP (Hyperlipidemia)**	44	35.8
**IHD (Ischemic heart disease)**	2	1.6
**HTN (Hypertension)**	79	64.2
**CVA (Cerebrovascular Accident)**	3	2.4
**Renal problems**	13	10.6
**Digestive problems**	3	2.4
**Pulmonary problems**	2	1.6
**Psychological problems**	2	1.6
**Fat kidney**	2	2.4
**Cancer**	3	2.4
**Thyroid problems**	5	4.1

The duration of admission was 7-154 days, with an average of 21.8 ± 24.96 days. The mortality rate was 11.4% (14 out of 123 patients suffering from postoperative nosocomial infections). There was a significant relationship between the death of patients and sepsis (patients with sepsis accounted for 64.3% of the deaths) (p-value = 0.000) (Table 5).

**Table 5:** The relationship between mortality rates of patients and the nosocomial infection in the cardiac surgery department.

Crosstab					
			Death		P-Value Chi-square
			Yes	No	
**Sepsis**	+	Count	9	9	0.000
		%	64.3%	8.3%	
	-	Count	5	100	
		%	35.7%	91.7%	
**SSI**	+	Count	2	25	0.365
		%	14.3%	22.9%	
	-	Count	12	84	
		%	85.7%	77.1%	
**UTI**	+	Count	2	20	0.55
		%	14.3%	18.3%	
	-	Count	12	89	
		%	85.7%	81.7%	
**SIRS**	+	Count	0	6	0.477
		%	0.0%	5.5%	
	-	Count	14	103	
		%	100.0%	94.5%	
**Catheter infection**	+	Count	1	5	0.52
		%	7.1%	4.6%	
	-	Count	13	104	
		%	92.9%	95.4%	
**Pneumonia**	+	Count	10	53	0.92
		% w	71.4%	48.6%	
	-	Count	4	56	
		%	28.6%	51.4%	

Note: SSI - Surgical site infection, UTI - Urinary tract infections, SIRS - systemic inflammatory response syndrome.

**Figure 2:**
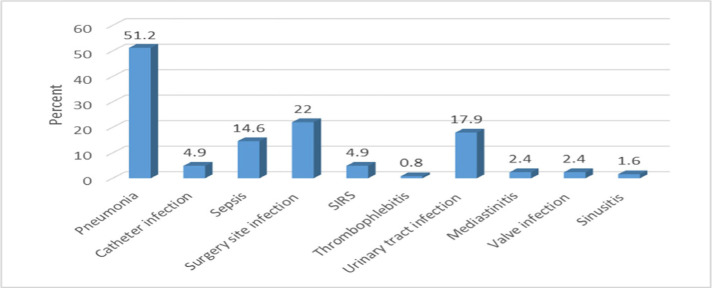
Frequency of various types of nosocomial infections in the subjects included in the study.

According to the results, out of a total of 610 cardiac surgeries in the Cardiac Surgery Department of the hospital, 123 suffered from microbial infection (20.16%). Pneumonia (51.2%), surgery site infection (22%), urinary tract infection (17.9%), and sepsis (14.6%) were the most common type of infections in the studied population. Systemic Inflammatory Response Syndrome (SIRS) (4.9%), catheter infections (4.9%), mediastinitis (2.4%), valve infection (2.4%) and thrombophlebitis (0.8%) were among the most frequent cases of known microbial infections (Figure 2).

Gram-positive cocci bacteria, Gram-negative bacilli and yeast were known to be the most common pathogens (30.8%, 28.4%, and 40.8%, respectively). Among the isolated Gram-positive cocci, 72.9% were coagulase-negative staphylococci and 27.1% were alpha-hemolytic streptococci. Acinetobacter (48.6%), Enterobacteriaceae (37.1%), and Pseudomonas aeruginosa (17.1%) constituted the Gram-negative bacilli.

The average duration of admission for patients with catheter infection was significantly higher than others (44.1 ± 60.5 days vs. 18.6 ± 23.1 days (p-value = 0.015). However, there was no relationship between other infections and the duration of admission (p-value > 0.05) (Table 6).

**Table 6:** The relationship between types of nosocomial infection and duration of admission.

		Mean	Std. Deviation	P-Value Mann Whitney
**Sepsis**	+	36.2222	35.04712	0.113
	-	23.0381	18.18649	
**SSI**	+	30.0370	23.62118	0.017
	-	23.5417	21.16448	
**SIRS**	+	17.3333	6.02218	0.298
	-	25.3590	22.24490	
**UTI**	+	20.6364	15.97184	0.055
	-	25.9109	22.82328	
**Pneumonia**	+	26.9048	24.51117	0.62
	-	22.9333	18.51053	
**Catheter infection**	+	60.5000	44.15767	0.015
	-	23.1453	18.62436	

Note: SSI - Surgical site infection, UTI - Urinary tract infections, SIRS - systemic inflammatory response syndrome.

## Discussion

Surgery site infection is one of the most common postoperative complications, which increases the prevalence of disease and mortality, duration of admission, and admission costs by 10-20% [11]. This occurs within 30 days after the surgery [12]. Also, cardiac surgery involves superficial and deep cutaneous/sternal infections, but also the site of saphenous vein removal a the level of the lower extremity [13]. Today, it is believed that most surgery site infections originate from the bacteria that enter the wound during surgery. It is also believed that surgical site infection occurs in the operating room [14]. So, this study aimed to investigate microbial contamination after cardiac surgery in a hospital cardiac surgery ward.

In our study, the incidence of microbial infections following cardiac surgery was estimated to be between 17% and 23%. Mazzeffi et al., Gelijns et al., and Larypoor reported that the prevalence of nosocomial infections following cardiac surgery was 4.65%, 15%, 5% and 28.7%, respectively [15-17]. The lower prevalence of infection in the studies of Gelijns et al. can be attributed to the types of infections. It should be noted that only major infections were investigated in these studies.

In our study, the mean age and duration of hospitalization of patients with catheter infections were significantly higher than other subjects, with a mean age of 72.33 ± 11.03 years. The mean duration of admission of patients with catheter infections was 44.1 ± 60.5 days versus 18.6 ± 23.1 days. However, there was no relationship between the age and duration of admission and other infections. Larypoor et al. found that there was a significant relationship between age and an increase in the prevalence of nosocomial infections. However, there was no relationship between gender and the prevalence of nosocomial infections [17].

In our study, Gram-positive cocci bacteria and Gram-negative bacilli were known to be the most common pathogens with a prevalence of 30.8% and 28.4%, respectively. Among isolated Gram-positive cocci isolates, 72.9% were coagulase-negative, and 27.1% were alpha-hemolytic streptococci. Acinetobacter (48.6%), Enterobacteriaceae (37.1%), and Pseudomonas aeruginosa (17.1%) constituted the Gram-negative bacilli. Hypertension (64.2%), coronary artery disease (61.8%), diabetes (43.9%) and hyperlipidemia (35.8%) were the most common diseases. The results of our study are similar to the results that Mazzeffi and his colleagues had in 2017 [15].

Pneumonia (51.2%), surgical site infections (22%), urinary tract infection (17.9%) and sepsis (14.6%) were the most common types of infection in the studied population. In contrast, Rostami et al. argued that surgery site infection and respiratory infections were the most commonly reported nosocomial infections [18].

Gelijns et al. examined major nosocomial infections following cardiac surgery. For this purpose, 5158 patients undergoing cardiac surgery were investigated within 65 days after surgery. According to the results, the overall prevalence of infection was about 5%, and there was no relationship between the prevalence of major infection and age, sex and BMI. The prevalence of infection in people with diabetes was significantly higher than others [6], and the results of this study were consistent with our results regarding some items.

Ibañez et al. showed that out of 2750 patients, 32 (1.2%) had pneumonia. Patients with pneumonia had a higher mortality rate than other people (28% versus 6.2%) (p-value <0.05). Pneumonia was also reported as a powerful tool for predicting mortality in patients undergoing cardiac surgery [19], and the results of this study were in line with our results.

## Conclusion

According to the results of this study, pneumonia is the most commonly reported microbial infection and ventilator-associated pneumonia is also high among the pneumonia cases. In general, it is necessary to take more serious measures to prevent and control these infections.

## Conflict of Interest

The authors declare that there is no conflict of interest.
